# 1-(3-Ammonio­methyl-2,4,6-trimethyl­benz­yl)-3-(2,4,6-trimethyl­phen­yl)imidazol-1-ium dibromide monohydrate

**DOI:** 10.1107/S1600536809012811

**Published:** 2009-04-10

**Authors:** Chao Zhang, Yong Ren, Mei-Ming Luo

**Affiliations:** a Key Laboratory of Green Chemistry and Technology of the Ministry of Education, College of Chemistry, Sichuan University, Chengdu 610064, People’s Republic of China; b Jiangsu Key Laboratory for Supramolecular Medicinal Materials and Applications, College of Life Science, Nanjing Normal University, No. 1 Wenyuan Road, Nanjing 210046, People’s Republic of China

## Abstract

In the title compound, C_25_H_35_N_3_^2+^·2Br^−^·H_2_O, the dihedral angles between the imidazole ring and the two outer benzene rings are 80.16 (16) and 69.40 (18)°. The component species are linked by N—H⋯Br, O—H⋯Br and C—H⋯Br hydrogen bonds.

## Related literature

For carbene ligands and complexes, see: Alcalde *et al.* (2007 ▶); Douthwaite *et al.* (2004 ▶); Magill *et al.* (2001 ▶). For phosphine ligands and complexes, see: Cao *et al.* (2000 ▶); Liou *et al.* (1995 ▶); Rybtchinski *et al.* (1996 ▶, 1999 ▶, 2001 ▶). For a related synthesis, see: Gandelman *et al.* (1997 ▶). For related literature, see: Caddick *et al.* (2004 ▶); Hahn (2006 ▶).
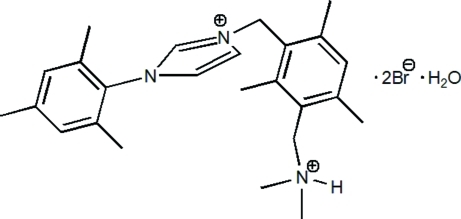


## Experimental

### 

#### Crystal data

C_25_H_35_N_3_^2+^·2Br^−^·H_2_O
*M* *_r_* = 555.40
Triclinic, 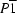

*a* = 10.594 (4) Å
*b* = 10.800 (3) Å
*c* = 13.193 (4) Å
α = 66.48 (4)°
β = 70.26 (4)°
γ = 80.58 (3)°
*V* = 1302.0 (9) Å^3^
*Z* = 2
Mo *K*α radiation
μ = 3.13 mm^−1^
*T* = 294 K
0.50 × 0.46 × 0.40 mm

#### Data collection

Enraf–Nonius CAD-4 diffractometer
Absorption correction: spherical (Farrugia, 1999 ▶) *T*_min_ = 0.303, *T*_max_ = 0.367
4856 measured reflections
4745 independent reflections
2966 reflections with *I* > 2σ(*I*)
*R*_int_ = 0.007
3 standard reflections every 300 reflections intensity decay: 2.7%

#### Refinement

*R*[*F* ^2^ > 2σ(*F* ^2^)] = 0.065
*wR*(*F* ^2^) = 0.203
*S* = 1.09
4745 reflections
300 parameters
4 restraints
H atoms treated by a mixture of independent and constrained refinement
Δρ_max_ = 1.01 e Å^−3^
Δρ_min_ = −0.58 e Å^−3^

### 

Data collection: *DIFRAC* (Gabe *et al*., 1993 ▶); cell refinement: *DIFRAC*; data reduction: *NRCVAX* (Gabe *et al*., 1989 ▶); program(s) used to solve structure: *SHELXS97* (Sheldrick, 2008 ▶); program(s) used to refine structure: *SHELXL97* (Sheldrick, 2008 ▶); molecular graphics: *ORTEP-3 for Windows* (Farrugia, 1999 ▶); software used to prepare material for publication: *SHELXL97*.

## Supplementary Material

Crystal structure: contains datablocks I, global. DOI: 10.1107/S1600536809012811/zl2182sup1.cif

Structure factors: contains datablocks I. DOI: 10.1107/S1600536809012811/zl2182Isup2.hkl

Additional supplementary materials:  crystallographic information; 3D view; checkCIF report

## Figures and Tables

**Table 1:** Hydrogen-bond geometry (Å, °)

*D*—H⋯*A*	*D*—H	H⋯*A*	*D*⋯*A*	*D*—H⋯*A*
O1—H1*O*1⋯Br1	0.81 (6)	2.58 (3)	3.355 (6)	160 (8)
O1—H1*O*2⋯Br2	0.82 (6)	2.49 (6)	3.283 (6)	165 (7)
N1—H1*N*⋯Br2	0.90 (5)	2.40 (3)	3.238 (5)	154 (6)
C9—H9*B*⋯Br2^i^	0.96	2.85	3.792 (7)	167
C12—H12*A*⋯Br2	0.96	2.82	3.765 (7)	168
C14—H14⋯Br1^ii^	0.93	2.79	3.436 (6)	128
C16—H16⋯Br2^iii^	0.93	2.81	3.597 (6)	143

Symmetry codes: (i) 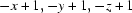; (ii) 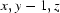; (iii) 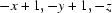.

**Fig. 1.**
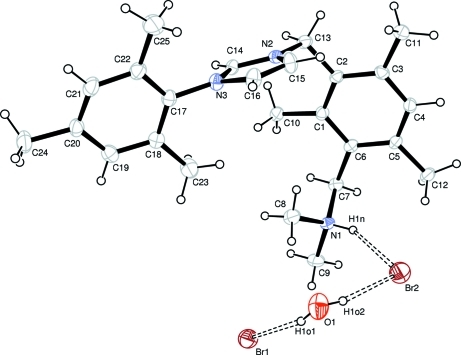
The molecular structure of the title compound with displacement ellipsoids drawn at the 30% probability level.

**Fig. 2.**
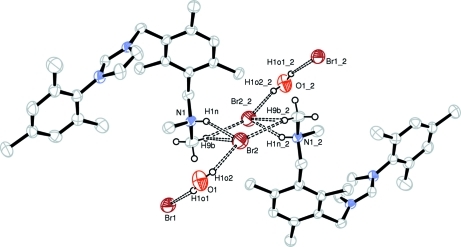
A partial packing diagram of the title compound. Hydrogen bonds are shown as dashed lines. H atoms not involved in hydrogen bonding are omitted.

## supplementary crystallographic information

### Comment

Transition metal complexes bearing N-heterocyclic carbene (NHC) ligands have attracted considerable attention in organometallic chemistry and catalysis. Compared to widely used phosphine complexes, the NHC complexes have been shown to be remarkably stable towards heat, air and moisture (Hahn, 2006). Rencently, phosphine complexes and phosphine-amido complexes were found to be active catalysts for the selective metal insertion into strong unstrained aryl-methyl bonds under very mild conditions (Cao *et al.*, 2000; Gandelman *et al.*, 1997; Liou *et al.*, 1995; Rybtchinski *et al.*, 1996; Rybtchinski *et al.*, 1999; Rybtchinski *et al.*, 2001). This prompted us to investigate whether NHC-amido complexes could provide different reactivity from phosphine-amido complexes in the aryl-methyl bond activation process. The title compound, a stable precursor of an NHC-amido ligand, was synthesized in moderate yield by reacting 1-[3-(bromomethyl)-2,4,6-trimethylbenzyl]-3-(2,4,6-trimethylphenyl)-1*H*-imidazol-3-ium bromide with a 10-fold excess of dimethylamine in methanol.

In the title compound (Fig. 1), the dihedral angles between the imidazole ring and the two outer benzene rings are 80.16 (16)° and 69.40 (18)°. A solvate water molecule and the bromide anions are linked to the main molecule *via *N—H···Br, O—H···Br and C—H···Br hydrogen bonds, and these intramolecular hydrogen bonds help to stabilize the crystal structure (Fig. 2).

### Experimental

A mixture of 1-mesityl-1*H*-imidazole (2.79 g, 15.0 mmol) and 2,6-bis(bromomethyl)mesitylene (4.82 g, 15.0 mmol) in dioxane (30 ml) was heated under reflex with stirring for 0.5 h. After this the mixture was cooled to room temperature, the white precipitate was filtered and washed with ether to remove unreacted starting material. The resulting mixture of the monoimidazolium (4.42 g, 59.9%) and diimidazolium salts was separated by flash chromatography (CH_2_Cl_2_/CH_3_OH (10/1, *v*/*v*)). Then, the monoimidazolium salt was dissovled in 35 ml of methanol and a 10-fold excess of dimethylamine (4.04 g, 89.8 mmol) was added. The resulting reaction mixture was heated for 6 h at 333 K. The product was obtained as a white powder (3.99 g, 82.8%) after the solvent removal under vacuum. Crystallization by slow evaporation of the solvent from a dichloromethane solution at ambient temperature afforded colorless crystals over a period of several days. m.p. 501 K. ^1^H NMR (400 MHz, CDCl_3_): δ = 10.68 (brs, 1H, N-*H*), 10.31 (s, 1H, Imi-*H*), 8.30 (s, 1H, Imi-*H*), 7.09 (s, 1H, Imi-*H*), 6.98 (m, 3H, Ar-*H*), 6.00 (s, 2H, Imi-C*H*_2_), 4.58 (s, 2H, NHC*H*_2_), 2.97 (s, 6H, NHC*H*_3_), 2.69 (s, 3H, ArC*H*_3_), 2.47 (s, 3H, ArC*H*_3_), 2.33 (s, 3H, ArC*H*_3_), 2.31 (s, 3H, ArC*H*_3_), 2.06 (s, 3H, ArC*H*_3_), 2.04 (s, 3H, ArC*H*_3_). ^13^C NMR (400 MHz, CDCl_3_): δ = 141.2, 140.7, 140.5, 139.9, 137.2, 134.1, 132.0, 130.7, 129.8, 128.5, 126.2, 123.9, 123.5, 56.1, 48.6, 21.2, 21.1, 20.2, 18.4, 17.6. MS (ESI) *m/z* = 455.5 [*M* - H_2_O - HBr]^+^_, _376.1 [*M* - H_2_O - HBr - Br]^+^_._

### Refinement

H atoms of the water molecule and N-H hydrogen atoms were located in difference Fourier maps and were refined isotropically with O-H and N-H distances of 0.82 (1) and 0.91 (1) Å, and their U_iso_ values were freely refined. All other H atoms were positioned geometrically, with C-H = 0.93, 0.96 and 0.97 Å for aromatic/imidazole, methyl and methylene H, respectively, and constrained to ride on their parent atoms with U_iso_(H) = xU_eq_(C), where x = 1.5 for methyl H and x = 1.2 for all other H atoms.

### Crystal data

**Table:**

C_25_H_35_N_3_^2+^·2Br^−^·H_2_O	*Z* = 2
*M**_r_* = 555.40	*F*(000) = 572
Triclinic, *P*1	*D*_x_ = 1.417 Mg m^−^^3^
Hall symbol: -P 1	Melting point: 501 K
*a* = 10.594 (4) Å	Mo *K*α radiation, λ = 0.71073 Å
*b* = 10.800 (3) Å	Cell parameters from 23 reflections
*c* = 13.193 (4) Å	θ = 4.5–7.8°
α = 66.48 (4)°	µ = 3.13 mm^−^^1^
β = 70.26 (4)°	*T* = 294 K
γ = 80.58 (3)°	Block, colourless
*V* = 1302.0 (9) Å^3^	0.50 × 0.46 × 0.40 mm

### Data collection

**Table:**

Enraf–Nonius CAD-4 diffractometer	2966 reflections with *I* > 2σ(*I*)
Radiation source: fine-focus sealed tube	*R*_int_ = 0.007
graphite	θ_max_ = 25.6°, θ_min_ = 1.8°
ω/2θ scans	*h* = −12→12
Absorption correction: for a sphere (Farrugia, 1999)	*k* = −4→13
*T*_min_ = 0.303, *T*_max_ = 0.367	*l* = −14→15
4856 measured reflections	3 standard reflections every 300 reflections
4745 independent reflections	intensity decay: 2.7%

### Refinement

**Table:**

Refinement on *F*^2^	Primary atom site location: structure-invariant direct methods
Least-squares matrix: full	Secondary atom site location: difference Fourier map
*R*[*F*^2^ > 2σ(*F*^2^)] = 0.065	Hydrogen site location: mixed
*wR*(*F*^2^) = 0.203	H atoms treated by a mixture of independent and constrained refinement
*S* = 1.09	*w* = 1/[σ^2^(*F*_o_^2^) + (0.12*P*)^2^ + 0.3033*P*] where *P* = (*F*_o_^2^ + 2*F*_c_^2^)/3
4745 reflections	(Δ/σ)_max_ < 0.001
300 parameters	Δρ_max_ = 1.01 e Å^−^^3^
4 restraints	Δρ_min_ = −0.58 e Å^−^^3^

### Special details

**Table:**

Geometry. All e.s.d.'s (except the e.s.d. in the dihedral angle between two l.s. planes) are estimated using the full covariance matrix. The cell e.s.d.'s are taken into account individually in the estimation of e.s.d.'s in distances, angles and torsion angles; correlations between e.s.d.'s in cell parameters are only used when they are defined by crystal symmetry. An approximate (isotropic) treatment of cell e.s.d.'s is used for estimating e.s.d.'s involving l.s. planes.
Refinement. Refinement of *F*^2^ against ALL reflections. The weighted *R*-factor *wR* and goodness of fit *S* are based on *F*^2^, conventional *R*-factors *R* are based on *F*, with *F* set to zero for negative *F*^2^. The threshold expression of *F*^2^ > σ(*F*^2^) is used only for calculating *R*-factors(gt) *etc*. and is not relevant to the choice of reflections for refinement. *R*-factors based on *F*^2^ are statistically about twice as large as those based on *F*, and *R*- factors based on ALL data will be even larger.

### Fractional atomic coordinates and isotropic or equivalent isotropic displacement parameters (Å^2^)

**Table:**

	*x*	*y*	*z*	*U*_iso_*/*U*_eq_	
Br1	0.05853 (7)	0.78890 (7)	0.34382 (7)	0.0653 (3)	
Br2	0.53218 (8)	0.62059 (8)	0.28647 (7)	0.0652 (3)	
O1	0.3386 (6)	0.7953 (7)	0.1251 (4)	0.0685 (14)	
H1O1	0.272 (4)	0.815 (8)	0.170 (4)	0.07 (3)*	
H1O2	0.397 (5)	0.764 (8)	0.157 (5)	0.07 (3)*	
N1	0.3013 (5)	0.4016 (4)	0.4497 (4)	0.0341 (10)	
H1N	0.383 (3)	0.438 (6)	0.413 (5)	0.056 (19)*	
N2	0.3043 (4)	0.0868 (4)	0.1764 (4)	0.0308 (9)	
N3	0.1566 (4)	0.2120 (4)	0.0972 (4)	0.0336 (10)	
C1	0.3488 (5)	0.1091 (5)	0.3941 (5)	0.0326 (11)	
C2	0.4293 (5)	0.0520 (5)	0.3132 (4)	0.0303 (11)	
C3	0.5697 (5)	0.0588 (5)	0.2778 (5)	0.0339 (12)	
C4	0.6263 (5)	0.1238 (5)	0.3225 (5)	0.0342 (12)	
H4	0.7193	0.1254	0.3010	0.041*	
C5	0.5505 (5)	0.1865 (5)	0.3978 (4)	0.0301 (11)	
C6	0.4093 (5)	0.1809 (5)	0.4329 (4)	0.0305 (11)	
C7	0.3254 (5)	0.2517 (5)	0.5130 (5)	0.0341 (12)	
H7A	0.3699	0.2408	0.5694	0.041*	
H7B	0.2395	0.2089	0.5548	0.041*	
C8	0.2255 (6)	0.4298 (6)	0.3664 (5)	0.0455 (14)	
H8A	0.2643	0.3772	0.3187	0.068*	
H8B	0.2294	0.5241	0.3183	0.068*	
H8C	0.1336	0.4064	0.4081	0.068*	
C9	0.2247 (6)	0.4663 (6)	0.5380 (6)	0.0473 (15)	
H9A	0.2131	0.5616	0.4984	0.071*	
H9B	0.2745	0.4511	0.5909	0.071*	
H9C	0.1384	0.4267	0.5802	0.071*	
C10	0.1998 (5)	0.0854 (6)	0.4431 (5)	0.0398 (13)	
H10A	0.1537	0.1555	0.3948	0.060*	
H10B	0.1678	0.0864	0.5201	0.060*	
H10C	0.1833	−0.0006	0.4455	0.060*	
C11	0.6620 (6)	−0.0024 (6)	0.1924 (5)	0.0444 (14)	
H11A	0.6464	−0.0971	0.2215	0.067*	
H11B	0.7537	0.0100	0.1826	0.067*	
H11C	0.6444	0.0413	0.1191	0.067*	
C12	0.6214 (6)	0.2566 (6)	0.4402 (5)	0.0385 (12)	
H12A	0.6090	0.3526	0.4046	0.058*	
H12B	0.7154	0.2326	0.4202	0.058*	
H12C	0.5850	0.2292	0.5229	0.058*	
C13	0.3650 (6)	−0.0154 (5)	0.2643 (5)	0.0351 (12)	
H13A	0.4318	−0.0707	0.2286	0.042*	
H13B	0.2960	−0.0741	0.3265	0.042*	
C14	0.1737 (5)	0.1112 (5)	0.1895 (4)	0.0302 (11)	
H14	0.1052	0.0644	0.2539	0.036*	
C15	0.3735 (6)	0.1725 (6)	0.0688 (5)	0.0421 (14)	
H15	0.4663	0.1750	0.0371	0.050*	
C16	0.2853 (6)	0.2512 (6)	0.0174 (5)	0.0437 (14)	
H16	0.3042	0.3187	−0.0562	0.052*	
C17	0.0284 (5)	0.2702 (5)	0.0807 (5)	0.0350 (12)	
C18	−0.0477 (5)	0.3458 (6)	0.1462 (5)	0.0378 (12)	
C19	−0.1741 (6)	0.3945 (6)	0.1334 (5)	0.0417 (13)	
H19	−0.2271	0.4449	0.1764	0.050*	
C20	−0.2221 (5)	0.3696 (6)	0.0589 (5)	0.0414 (13)	
C21	−0.1385 (6)	0.3010 (6)	−0.0093 (6)	0.0489 (15)	
H21	−0.1682	0.2895	−0.0636	0.059*	
C22	−0.0132 (6)	0.2492 (6)	−0.0001 (5)	0.0423 (13)	
C23	0.0000 (7)	0.3743 (7)	0.2297 (6)	0.0521 (16)	
H23A	−0.0364	0.4603	0.2334	0.078*	
H23B	0.0963	0.3757	0.2037	0.078*	
H23C	−0.0292	0.3050	0.3052	0.078*	
C24	−0.3623 (7)	0.4168 (8)	0.0498 (8)	0.066 (2)	
H24A	−0.4166	0.4323	0.1187	0.099*	
H24B	−0.4016	0.3488	0.0413	0.099*	
H24C	−0.3575	0.4993	−0.0164	0.099*	
C25	0.0750 (8)	0.1756 (9)	−0.0766 (8)	0.073 (2)	
H25A	0.0258	0.1644	−0.1215	0.109*	
H25B	0.1022	0.0886	−0.0293	0.109*	
H25C	0.1529	0.2271	−0.1278	0.109*	

### Atomic displacement parameters (Å^2^)

**Table:**

	*U*^11^	*U*^22^	*U*^33^	*U*^12^	*U*^13^	*U*^23^
Br1	0.0458 (4)	0.0520 (4)	0.0827 (6)	−0.0118 (3)	0.0013 (3)	−0.0227 (4)
Br2	0.0657 (5)	0.0598 (5)	0.0600 (5)	−0.0090 (3)	−0.0138 (4)	−0.0137 (4)
O1	0.058 (3)	0.106 (4)	0.053 (3)	−0.005 (3)	−0.016 (3)	−0.039 (3)
N1	0.036 (2)	0.034 (2)	0.040 (3)	−0.0018 (19)	−0.012 (2)	−0.020 (2)
N2	0.037 (2)	0.028 (2)	0.032 (2)	0.0007 (18)	−0.0126 (19)	−0.0136 (19)
N3	0.033 (2)	0.034 (2)	0.030 (2)	−0.0029 (18)	−0.0117 (19)	−0.0063 (19)
C1	0.031 (3)	0.030 (3)	0.034 (3)	−0.004 (2)	−0.010 (2)	−0.009 (2)
C2	0.035 (3)	0.024 (2)	0.033 (3)	−0.004 (2)	−0.012 (2)	−0.008 (2)
C3	0.036 (3)	0.026 (3)	0.037 (3)	0.003 (2)	−0.011 (2)	−0.009 (2)
C4	0.028 (3)	0.030 (3)	0.042 (3)	−0.003 (2)	−0.012 (2)	−0.009 (2)
C5	0.037 (3)	0.022 (2)	0.033 (3)	−0.004 (2)	−0.019 (2)	−0.003 (2)
C6	0.037 (3)	0.028 (3)	0.027 (3)	−0.006 (2)	−0.011 (2)	−0.007 (2)
C7	0.042 (3)	0.032 (3)	0.031 (3)	−0.001 (2)	−0.014 (2)	−0.011 (2)
C8	0.061 (4)	0.035 (3)	0.050 (4)	0.004 (3)	−0.036 (3)	−0.012 (3)
C9	0.051 (3)	0.044 (3)	0.056 (4)	0.004 (3)	−0.011 (3)	−0.033 (3)
C10	0.034 (3)	0.047 (3)	0.048 (3)	0.000 (2)	−0.013 (3)	−0.027 (3)
C11	0.037 (3)	0.050 (3)	0.053 (4)	0.000 (3)	−0.009 (3)	−0.030 (3)
C12	0.042 (3)	0.034 (3)	0.046 (3)	−0.007 (2)	−0.018 (3)	−0.015 (3)
C13	0.043 (3)	0.027 (3)	0.040 (3)	0.001 (2)	−0.020 (2)	−0.013 (2)
C14	0.027 (3)	0.033 (3)	0.029 (3)	−0.007 (2)	−0.007 (2)	−0.009 (2)
C15	0.029 (3)	0.052 (3)	0.033 (3)	−0.004 (3)	−0.005 (2)	−0.007 (3)
C16	0.037 (3)	0.052 (3)	0.033 (3)	−0.012 (3)	−0.008 (2)	−0.005 (3)
C17	0.033 (3)	0.036 (3)	0.033 (3)	−0.005 (2)	−0.010 (2)	−0.009 (2)
C18	0.039 (3)	0.037 (3)	0.033 (3)	−0.004 (2)	−0.011 (2)	−0.009 (2)
C19	0.040 (3)	0.037 (3)	0.039 (3)	0.001 (2)	−0.010 (3)	−0.008 (3)
C20	0.034 (3)	0.036 (3)	0.051 (4)	−0.006 (2)	−0.017 (3)	−0.007 (3)
C21	0.054 (4)	0.047 (3)	0.056 (4)	−0.004 (3)	−0.034 (3)	−0.013 (3)
C22	0.045 (3)	0.043 (3)	0.045 (3)	−0.003 (3)	−0.019 (3)	−0.017 (3)
C23	0.050 (4)	0.067 (4)	0.045 (4)	0.007 (3)	−0.018 (3)	−0.027 (3)
C24	0.049 (4)	0.057 (4)	0.099 (6)	0.004 (3)	−0.044 (4)	−0.021 (4)
C25	0.075 (5)	0.090 (6)	0.084 (6)	0.022 (4)	−0.042 (4)	−0.059 (5)

### Geometric parameters (Å, °)

**Table:**

O1—H1O1	0.81 (6)	C10—H10C	0.9600
O1—H1O2	0.82 (6)	C11—H11A	0.9600
N1—C8	1.482 (7)	C11—H11B	0.9600
N1—C7	1.521 (7)	C11—H11C	0.9600
N1—C9	1.526 (7)	C12—H12A	0.9600
N1—H1N	0.90 (5)	C12—H12B	0.9600
N2—C14	1.330 (6)	C12—H12C	0.9600
N2—C15	1.371 (7)	C13—H13A	0.9700
N2—C13	1.492 (7)	C13—H13B	0.9700
N3—C14	1.314 (6)	C14—H14	0.9300
N3—C16	1.418 (7)	C15—C16	1.328 (8)
N3—C17	1.449 (7)	C15—H15	0.9300
C1—C6	1.405 (7)	C16—H16	0.9300
C1—C2	1.409 (7)	C17—C22	1.387 (8)
C1—C10	1.512 (7)	C17—C18	1.393 (8)
C2—C3	1.406 (7)	C18—C19	1.396 (8)
C2—C13	1.497 (7)	C18—C23	1.503 (8)
C3—C4	1.380 (8)	C19—C20	1.376 (9)
C3—C11	1.517 (7)	C19—H19	0.9300
C4—C5	1.382 (7)	C20—C21	1.386 (9)
C4—H4	0.9300	C20—C24	1.518 (8)
C5—C6	1.412 (7)	C21—C22	1.379 (8)
C5—C12	1.503 (7)	C21—H21	0.9300
C6—C7	1.510 (7)	C22—C25	1.511 (9)
C7—H7A	0.9700	C23—H23A	0.9600
C7—H7B	0.9700	C23—H23B	0.9600
C8—H8A	0.9600	C23—H23C	0.9600
C8—H8B	0.9600	C24—H24A	0.9600
C8—H8C	0.9600	C24—H24B	0.9600
C9—H9A	0.9600	C24—H24C	0.9600
C9—H9B	0.9600	C25—H25A	0.9600
C9—H9C	0.9600	C25—H25B	0.9600
C10—H10A	0.9600	C25—H25C	0.9600
C10—H10B	0.9600		
			
H1O1—O1—H1O2	109 (6)	H11A—C11—H11C	109.5
C8—N1—C7	113.5 (4)	H11B—C11—H11C	109.5
C8—N1—C9	108.8 (4)	C5—C12—H12A	109.5
C7—N1—C9	109.4 (4)	C5—C12—H12B	109.5
C8—N1—H1N	111 (4)	H12A—C12—H12B	109.5
C7—N1—H1N	107 (4)	C5—C12—H12C	109.5
C9—N1—H1N	108 (4)	H12A—C12—H12C	109.5
C14—N2—C15	108.3 (4)	H12B—C12—H12C	109.5
C14—N2—C13	125.8 (4)	N2—C13—C2	110.9 (4)
C15—N2—C13	125.9 (4)	N2—C13—H13A	109.5
C14—N3—C16	107.7 (4)	C2—C13—H13A	109.5
C14—N3—C17	125.5 (4)	N2—C13—H13B	109.5
C16—N3—C17	126.7 (4)	C2—C13—H13B	109.5
C6—C1—C2	119.5 (5)	H13A—C13—H13B	108.1
C6—C1—C10	120.9 (5)	N3—C14—N2	109.3 (4)
C2—C1—C10	119.5 (5)	N3—C14—H14	125.3
C3—C2—C1	120.1 (5)	N2—C14—H14	125.3
C3—C2—C13	120.0 (5)	C16—C15—N2	108.4 (5)
C1—C2—C13	119.9 (5)	C16—C15—H15	125.8
C4—C3—C2	118.8 (5)	N2—C15—H15	125.8
C4—C3—C11	118.5 (5)	C15—C16—N3	106.2 (5)
C2—C3—C11	122.7 (5)	C15—C16—H16	126.9
C3—C4—C5	122.7 (5)	N3—C16—H16	126.9
C3—C4—H4	118.6	C22—C17—C18	122.7 (5)
C5—C4—H4	118.6	C22—C17—N3	118.7 (5)
C4—C5—C6	118.7 (5)	C18—C17—N3	118.6 (5)
C4—C5—C12	118.8 (5)	C17—C18—C19	117.4 (5)
C6—C5—C12	122.6 (5)	C17—C18—C23	122.8 (5)
C1—C6—C5	120.0 (4)	C19—C18—C23	119.7 (5)
C1—C6—C7	120.9 (5)	C20—C19—C18	121.6 (5)
C5—C6—C7	119.1 (4)	C20—C19—H19	119.2
C6—C7—N1	113.2 (4)	C18—C19—H19	119.2
C6—C7—H7A	108.9	C19—C20—C21	118.3 (5)
N1—C7—H7A	108.9	C19—C20—C24	121.4 (6)
C6—C7—H7B	108.9	C21—C20—C24	120.4 (6)
N1—C7—H7B	108.9	C22—C21—C20	122.9 (6)
H7A—C7—H7B	107.8	C22—C21—H21	118.6
N1—C8—H8A	109.5	C20—C21—H21	118.6
N1—C8—H8B	109.5	C21—C22—C17	116.9 (5)
H8A—C8—H8B	109.5	C21—C22—C25	121.1 (6)
N1—C8—H8C	109.5	C17—C22—C25	122.0 (5)
H8A—C8—H8C	109.5	C18—C23—H23A	109.5
H8B—C8—H8C	109.5	C18—C23—H23B	109.5
N1—C9—H9A	109.5	H23A—C23—H23B	109.5
N1—C9—H9B	109.5	C18—C23—H23C	109.5
H9A—C9—H9B	109.5	H23A—C23—H23C	109.5
N1—C9—H9C	109.5	H23B—C23—H23C	109.5
H9A—C9—H9C	109.5	C20—C24—H24A	109.5
H9B—C9—H9C	109.5	C20—C24—H24B	109.5
C1—C10—H10A	109.5	H24A—C24—H24B	109.5
C1—C10—H10B	109.5	C20—C24—H24C	109.5
H10A—C10—H10B	109.5	H24A—C24—H24C	109.5
C1—C10—H10C	109.5	H24B—C24—H24C	109.5
H10A—C10—H10C	109.5	C22—C25—H25A	109.5
H10B—C10—H10C	109.5	C22—C25—H25B	109.5
C3—C11—H11A	109.5	H25A—C25—H25B	109.5
C3—C11—H11B	109.5	C22—C25—H25C	109.5
H11A—C11—H11B	109.5	H25A—C25—H25C	109.5
C3—C11—H11C	109.5	H25B—C25—H25C	109.5
			
C6—C1—C2—C3	4.4 (7)	C17—N3—C14—N2	−178.2 (5)
C10—C1—C2—C3	−172.1 (5)	C15—N2—C14—N3	−2.5 (6)
C6—C1—C2—C13	−175.3 (5)	C13—N2—C14—N3	176.9 (4)
C10—C1—C2—C13	8.2 (7)	C14—N2—C15—C16	1.5 (6)
C1—C2—C3—C4	−0.9 (7)	C13—N2—C15—C16	−177.9 (5)
C13—C2—C3—C4	178.8 (5)	N2—C15—C16—N3	0.0 (7)
C1—C2—C3—C11	179.1 (5)	C14—N3—C16—C15	−1.5 (6)
C13—C2—C3—C11	−1.3 (8)	C17—N3—C16—C15	179.2 (5)
C2—C3—C4—C5	−2.2 (8)	C14—N3—C17—C22	−110.0 (6)
C11—C3—C4—C5	177.8 (5)	C16—N3—C17—C22	69.2 (7)
C3—C4—C5—C6	1.7 (8)	C14—N3—C17—C18	70.5 (7)
C3—C4—C5—C12	−179.0 (5)	C16—N3—C17—C18	−110.3 (6)
C2—C1—C6—C5	−4.9 (7)	C22—C17—C18—C19	4.0 (8)
C10—C1—C6—C5	171.5 (5)	N3—C17—C18—C19	−176.5 (5)
C2—C1—C6—C7	175.7 (4)	C22—C17—C18—C23	−177.0 (6)
C10—C1—C6—C7	−7.9 (8)	N3—C17—C18—C23	2.5 (8)
C4—C5—C6—C1	1.9 (7)	C17—C18—C19—C20	−0.3 (8)
C12—C5—C6—C1	−177.4 (5)	C23—C18—C19—C20	−179.3 (6)
C4—C5—C6—C7	−178.6 (5)	C18—C19—C20—C21	−3.7 (9)
C12—C5—C6—C7	2.1 (7)	C18—C19—C20—C24	176.9 (6)
C1—C6—C7—N1	−97.4 (6)	C19—C20—C21—C22	4.4 (9)
C5—C6—C7—N1	83.1 (6)	C24—C20—C21—C22	−176.3 (6)
C8—N1—C7—C6	62.4 (6)	C20—C21—C22—C17	−0.9 (9)
C9—N1—C7—C6	−175.9 (4)	C20—C21—C22—C25	−179.7 (7)
C14—N2—C13—C2	−109.4 (6)	C18—C17—C22—C21	−3.4 (9)
C15—N2—C13—C2	69.9 (6)	N3—C17—C22—C21	177.1 (5)
C3—C2—C13—N2	−104.4 (5)	C18—C17—C22—C25	175.4 (6)
C1—C2—C13—N2	75.3 (6)	N3—C17—C22—C25	−4.1 (9)
C16—N3—C14—N2	2.5 (6)		

### Hydrogen-bond geometry (Å, °)

**Table:**

*D*—H···*A*	*D*—H	H···*A*	*D*···*A*	*D*—H···*A*
O1—H1O1···Br1	0.81 (6)	2.58 (3)	3.355 (6)	160 (8)
O1—H1O2···Br2	0.82 (6)	2.49 (6)	3.283 (6)	165 (7)
N1—H1N···Br2	0.90 (5)	2.40 (3)	3.238 (5)	154 (6)
C9—H9B···Br2^i^	0.96	2.85	3.792 (7)	167
C12—H12A···Br2	0.96	2.82	3.765 (7)	168
C14—H14···Br1^ii^	0.93	2.79	3.436 (6)	128
C16—H16···Br2^iii^	0.93	2.81	3.597 (6)	143

Symmetry codes: (i) −*x*+1, −*y*+1, −*z*+1; (ii) *x*, *y*−1, *z*; (iii) −*x*+1, −*y*+1, −*z*.
